# Selective depletion of tumour suppressors Deleted in Colorectal Cancer (DCC) and neogenin by environmental and endogenous serine proteases: linking diet and cancer

**DOI:** 10.1186/s12885-016-2795-y

**Published:** 2016-10-06

**Authors:** Caroline M. Forrest, Kara McNair, Maria C. J. Vincenten, L. Gail Darlington, Trevor W. Stone

**Affiliations:** 1 College of Medical, Veterinary and Life Sciences, West Medical Building, University of Glasgow, Glasgow, G12 8QQ UK; 2 Internal Medicine, Ashtead Hospital, Ashtead, Surrey KT21 2SB UK

**Keywords:** Deleted in colorectal cancer, DCC, Neogenin, Serine proteases, Chymotrypsin, Subtilisin

## Abstract

**Background:**

The related tumour suppressor proteins Deleted in Colorectal Cancer (DCC) and neogenin are absent or weakly expressed in many cancers, whereas their insertion into cells suppresses oncogenic behaviour. Serine proteases influence the initiation and progression of cancers although the mechanisms are unknown.

**Methods:**

The effects of environmental (bacterial subtilisin) and endogenous mammalian (chymotrypsin) serine proteases were examined on protein expression in fresh, normal tissue and human neuroblastoma and mammary adenocarcinoma lines. Cell proliferation and migration assays (chemoattraction and wound closure) were used to examine cell function. Cells lacking DCC were transfected with an ectopic *dcc* plasmid.

**Results:**

Subtilisin and chymotrypsin selectively depleted DCC and neogenin from cells at nanomolar concentrations without affecting related proteins. Cells showed reduced adherence and increased migration, but after washing they re-attached within 24 h, with recovery of protein expression. These effects are induced by chymotryptic activity as they are prevented by chymostatin and the soybean Bowman-Birk inhibitor typical of many plant protease inhibitors.

**Conclusions:**

*Bacillus subtilis*, which secretes subtilisin is widely present in soil, the environment and the intestinal contents, while subtilisin itself is used in meat processing, animal feed probiotics and many household cleaning agents. With chymotrypsin present in chyme, blood and tissues, these proteases may contribute to cancer development by depleting DCC and neogenin. Blocking their activity by Bowman-Birk inhibitors may explain the protective effects of a plant diet. Our findings identify a potential non-genetic contribution to cancer cell behaviour which may explain both the association of processed meats and other factors with cancer incidence and the protection afforded by plant-rich diets, with significant implications for cancer prevention.

**Electronic supplementary material:**

The online version of this article (doi:10.1186/s12885-016-2795-y) contains supplementary material, which is available to authorized users.

## Background

Expression of the tumour suppressor proteins Deleted in Colorectal Cancer (DCC) or the structurally related protein neogenin is reduced in many cancers, the extent of loss compared with control tissue correlating with degree of metastasis and with poor patient prognosis [1–8]. Although early work on DCC revealed genetic abnormalities such as frequent Loss of Heterozygosity, outright mutations have been encountered less frequently than expected, leading to suggestions that functional abnormalities of the protein may often be non-genetic in origin [9, 10]. Experimental inhibition of DCC can increase proliferation or migration in different cell types [11–14] while, conversely, insertion of the *dcc* gene into cells inhibits proliferation, invasion and metastatic potential [13–17].

Similarly, low levels of the structurally related protein neogenin have been linked with an increased propensity to develop cancer [18–22], while over-expression induces apoptosis [22, 23]. Reduced neogenin expression has a particularly prominent association with cancers in the CNS and mammary tissues [21, 24–26].

Both DCC and neogenin are involved in defining the balance between cell survival or death and between differentiation and de-differentiation towards an un-regulated, hyper-proliferative and potentially oncogenic phenotype [27–29]. They are receptors for the ligand netrin, an extracellular, secreted protein. In the absence of netrin, DCC or neogenin activate cell death programmes including apoptosis, leading to the concept that they are “dependence receptors”, regulating cell viability depending on the ambient concentration of netrin [30–33]. If cells escape from their home tissue by damage, inflammation or natural turnover, the reduced netrin concentration unleashes dependence receptor-induced cell death, preventing uncontrolled proliferation in distant tissues. If DCC or neogenin are absent, however, this mechanism cannot operate and proliferation or migration will proceed unchecked [34].

Serine proteases are present in relatively high concentrations in many cancers and can influence cell proliferation and migration [27, 28, 35–41] while serine protease inhibitors can suppress tumour invasion and metastasis [42–45]. Specific sites and mechanisms of action, however, remain unclear.

We now report a major link between these two groups of compounds, showing that nanomolar concentrations of the serine protease subtilisin, a chymotryptic protease secreted by the common environmental bacterium *Bacillus subtilis* and related organisms, and mammalian chymotrypsin itself, deplete the levels of DCC and neogenin in cells. Expression of a third dependence receptor targeted by netrin, unco-ordinated-5C [46], is also affected but to a lesser degree than DCC or neogenin.

*B. subtilis* is present in soil, while subtilisin itself is used to increase tenderness and flavour in some processed meat products and is present in many cleaning materials. Since orally acquired live bacteria and spores of *B. subtilis* can survive in the intestine of humans and other mammals [47], and the concentrations of chymotrypsin in tissues and intestinal chyme are similar to those studied here, their ability to remove DCC and neogenin could represent a significant factor in the effects of diet and environment on cancer susceptibility.

We also show that Bowman-Birk inhibitors present in many food crops including fruit, vegetables and cereals [48–51] can block these effects of serine proteases, providing a potential explanation of the protective effects of a plant-rich diet. The removal or reduction of subtilisin in the human food chain and cleaning products, and a plant-based diet rich in Bowman-Birk inhibitors, might substantially reduce the worldwide incidence of several forms of cancer.

## Methods

### Tissue slices

Initial experiments were performed using sections of adult rat hippocampus which can be maintained for several hours without the need for serum or other additives. These slices are exactly similar to those used routinely for the electrophysiological recording of synaptic potentials [52, 53]. Briefly, male Wistar rats (100-150 g from Harlan Olac, UK) were killed using urethane (5 ml/kg) and cervical dislocation. The brain was removed into ice-cold artificial cerebrospinal fluid (aCSF) of composition (in mM): NaCl 115; KH_2_PO_4_ 2.2; KCl 2; MgSO_4_ 1.2; NaHCO_3_ 25; CaCl_2_ 2.5; glucose 10, gassed with 5 % CO_2_ in air. The hippocampi were chopped into 450μm transverse slices and allowed to recover for 1-2 h, when compounds were added for 4 h.

### Immunoblotting

Western blots were generated as described previously [52, 54, 55]. Briefly, tissue slices were homogenised in RIPA buffer with a Roche complete protease inhibitor tablet and centrifuged (18000 *g,* 5 min, 4 °C). Supernatant protein concentration was determined using the Bio-Rad assay (Bio-Rad, Hemel Hempstead, UK) and normalised to 10 μg. The protein samples were subsequently loaded onto NuPAGE Novex 4–12 % Bis-Tris (1.0 mm) gels and run at 175 volts for 70 min. The separated proteins were blotted onto Invitrolon polyvinylidene difluoride membranes (35 V, 75 min) after which membranes were rinsed, blocked for 1 h in a milk solution in Tris-buffered saline containing 0.05 % Tween (TBST), followed by overnight incubation at 4 °C with primary antibody. After further washing and treatment with horseradish peroxidase (HRP) conjugated secondary antibody, blots were washed 3 times and visualised using a Pierce Enhanced Chemiluminescence 2 detection kit.

Western blot analysis was carried out using the following primary antibodies:-

*From Santa Cruz, Insight Biotechnology, Wembley, UK*:- Neogenin (goat polyclonal, sc-6536, 1:1000 dilution); Unc5A (goat polyclonal, sc-67902, 1:1000 dilution); Unc5C (goat polyclonal, sc-54442 1:500 dilution); Shh (goat polyclonal, sc-1194, 1:1000 dilution); RhoA (mouse monoclonal, sc-418, 1:5000 dilution); *From BD Pharmingen, Oxford, UK):-* DCC (mouse monoclonal, 554223, 1:5000 dilution).

RhoA was included in all experiments as the standard loading and transfer control since in motile and invasive cells the classical controls such as actin and tubulin, associated with the cytoskeletal involvement in movement, are inappropriate [56, 57].

Secondary HRP-conjugated antibodies were used at a 1:5000 dilution: donkey anti-goat HRP (sc-2020), goat anti-mouse (sc-2005), and donkey anti-rabbit HRP (sc-2313) (Santa Cruz, Insight Biotechnology, Wembley, UK). The blots were quantified using Image J [54, 55, 58].

### Immunocytochemistry

Cells were passaged into 24-well plates containing poly D-lysine (50 μg/ml) coated glass coverslips and after experimental observation were fixed with 4 % paraformaldehyde (PFA), rinsed and incubated overnight in primary antibody. After further rinsing with PBS, cells were incubated in the appropriate secondary fluorescent antibody at 1:200 dilution in PBS with 0.3 % Triton X (1 h) followed by rinsing and mounting the coverslips with Vectashield® fluorescent mounting medium.

Primary antibodies used were: DCC 1:500 (BD Pharmingen, Oxford, UK, #554223); doublecortin (DCX) 1:200 (Santa Cruz, California; #SC8066), neogenin 1:500 (Santa Cruz, California; #SC6536).

Secondary antibodies (all 1:200) were AlexaFluor 594 goat anti-mouse #A11032; AlexaFluor 488 goat anti-rabbit #A11008; AlexaFluor rabbit anti-goat #A11078 (Life Technologies, Paisley, UK).

### Cell cultures

All cells were maintained using the procedures and media recommended by the supplier ECACC (Wiltshire, UK). The SH-SY5Y cell line, #94030304) is an adherent, human neuroblastoma cell line obtained at a passage number of 17. This cell line was only used up to passage 30 as beyond this the cells lose their neuronal characteristics. Cells were plated at an initial density of 5 × 10^4^ cells/ml unless otherwise stated. This cell line was passaged once per week and fed every 2–3 days with a 50 % media change.

The MDA-MB-231 cell line is an adherent, human Caucasian breast adenocarcinoma cell line purchased at passage 40 (ECACC #92020424) and used for no more than 30 further passages. Cells were plated at an initial density of 1 × 10^5^ cells/ml unless otherwise stated and were passaged twice per week and fed every 2–3 days, when required, with a 50 % media change.

The MCF-7 cell line is an adherent, human Caucasian breast adenocarcinoma cell line purchased at passage 15 (ECACC #86012803) and used for no more than 30 passages following resuscitation. Cells were plated at an initial density of 1 × 10^5^ cells/ml unless otherwise stated. These cells were passaged once per week and fed every 2–3 days with a 50 % medium change.

Human Caucasian colon adenocarcinoma (CaCo-2) cells (ECACC #86010202) were purchased at passage number 45 and were used for up to 20 passages. Cells were plated at an initial density of 1 × 10^5^ cells/ml unless otherwise stated and were passaged once per week and fed every 2–3 days with a 50 % media change.

### Replating and recovery

MDA-MB-231 cells were plated at a density of 3x10^5^ cells/ml in 6-well plates and were allowed to attach to the plates for 24 h before the addition of subtilisin (1 μM). After 24 h, detached cells were collected, washed and replated in fresh medium, then left for 24 h before harvesting for Western blotting. Cells were photographed at x20 magnification on an Olympus IX50 inverted microscope attached to an Olympus DP50 camera.

### Agarose spot migration

Based on a published method [59], low melting point agarose was prepared and human netrin-4 (R & D systems, #1254-N4) was added to 1000 ng/ml. A spot of 10 μl of LMA solution was pipetted into the centre of each well and allowed to set for at least 1 h at 4 °C. Cells were passaged and transferred to the spot-containing wells for 4 h at 37 °C, when the medium was changed to contain 0.1 % serum. After 24 h, six photographs were taken of each well at x4 magnification, with a minimum of 4 different experiments from 4 different passages. Image J (http://rsb.info.nih.gov/ij/) was used to analyse cell distribution in the photographs and the average number of cells per spot was calculated for each experiment.

### Wound healing assay

Cells were plated at a density of 2 × 10^5^ cells/ml in 6-well plates which had been marked with a reference grid. A confluent monolayer formed in approximately 3 days, when a scratch wound was made down the centre of each well [60, 61]. The cells were then washed and placed in normal (10 % serum) medium. Photographs (x4 magnification) were taken immediately (0 h) and at 72 h at 3 points along the wound using an Olympus DP50 camera attached to an Olympus IX50 inverted microscope. The score grid ensured that the same area of cells was captured in each photograph. Experimental treatments were started after the initial photograph, and wound closure was quantified by measuring open wound area, using T-scratch software [62]. When subtilisin was added, photographs were also taken at 24 h and 48 h.

### Morphology of SH-SY5Y cells

To examine morphological changes in SH-SY5Y cells, passaged cells were grown in medium containing 1 % FCS for 3 days to induce neurite formation. Chymotrypsin (100nM) was added on days 1 and 3. On day 6, cells were rinsed, fixed with 4 % paraformaldehyde and dehydrated followed by staining with filtered Cresyl Violet for 10 min. From three experiments 300 cells were examined, using 50 control cells and 50 treated cells from each group. Cells were photographed (x20) and the images were subsequently analysed using Image J [63] to determine the number and length of neurites per cell, the number of neurite branch points and the soma diameter.

### Proliferation (bromodeoxyuridine BrdU assay)

Proliferation was assessed in the MCF-7 cells using a colorimetric BrdU ELISA kit (ab126556, Abcam, Cambridge, UK). Each assay was performed in triplicate. MCF-7 cells were plated at 2x10^5^ cells/ml in 96-well plates and left to attach for 24 h before treatment with subtilisin (30, 100 and 300nM) or α-chymotrypsin (300 and 1000nM) for 24 h. BrdU reagent was added for the final 2, 6 and 24 h of incubation after which the cells were fixed and the DNA denatured. Briefly, cells were washed, incubated with detector antibody (1 h), washed again and incubated with peroxidase conjugated secondary antibody (1 h). After washing, cells were incubated with TMB peroxidase substrate and a stop solution was added before reading in a microplate reader (using a dual wavelength of 450/550 nm). Proliferation was expressed as the mean optical density of BrdU positively labelled cells/BrdU negative cells.

### Transfection of DCC plasmid

A pCMV DCC plasmid (#16459) was obtained from Addgene courtesy of Dr. B. Vogelstein. This plasmid contains the Cytomegalovirus (CMV) promoter, a strong constitutive promoter, the DCC gene flanked by 2 XhoI restriction sites, polyadenylation signal (Poly A), Ampicillin resistance gene and Neomycin resistance gene. DH5alpha *E.coli* cells containing the plasmid were grown on agar plates containing 75 μg/ml ampicillin. Individual colonies were inoculated into Luria-Bertani medium containing ampicillin and grown overnight. The remaining bacterial medium was used to extract pCMV DCC plasmid DNA using Pure yield plasmid miniprep system (A1223, Promega UK) following manufacturer’s instructions. Transfection was performed in SH-SY5Y and MCF-7 cells passaged 24 h prior to transfection. To promote the efficiency of transfection, cells were plated at a density sufficient to result in approximately 60–70 % confluency 24 h after passage. At this time, the medium was removed from each well and replaced with antibiotic free medium for approximately 1 h. The transfection reagent Lipofectamine 2000 was used for successful transfection of the DCC plasmid according to the manufacturer’s instructions using a ratio of 1:1 DNA: Lipofectamine 2000. Briefly, DNA (1000 ng) was combined with Lipofectamine 2000 in the presence of Optimem and then incubated at room temperature for 15 min before adding to cells in antibiotic-free medium. Approximately 4–5 h after the introduction of DNA, medium was removed and replaced with medium containing antibiotics and test agents as required. Cells were either harvested for Western blot analysis or used for further experiments 48 h after the initial introduction of the DCC plasmid.

### Data analysis and statistics

Comparisons between two experimental samples were made using a two-tailed Student’s *t* test. Statistical comparisons were made between groups of samples using ANOVA followed by the Dunnet *post hoc* test to compare several datasets with a common control or the Bonferroni *post hoc* multiple comparison test for selected datasets, using Instat 3.0 software. A probability value of 0.05 was adopted as the criterion for significance.

## Results

In addition to DCC and neogenin several proteins have been examined in this study that are functionally related to them but which are structurally dissimilar. This comparison allowed assessment of the selectivity of the proteases on proteins and pathways which interact with DCC or neogenin. These proteins include unco-ordinated-5C (Unc-5C), another dependence receptor for netrin which can complex with DCC and which is absent from a variety of cancers [64–67]; Unc-5A: to assess the selectivity of serine proteases for unc5 family members; the small GTPase enzyme RhoA [68, 69]; Sonic hedgehog (Shh), a secreted protein involved in embryonic morphogenesis, cell location and polarisation [70, 71] and which modulates the expression of neogenin and netrin [72].

**Fig. 1:**
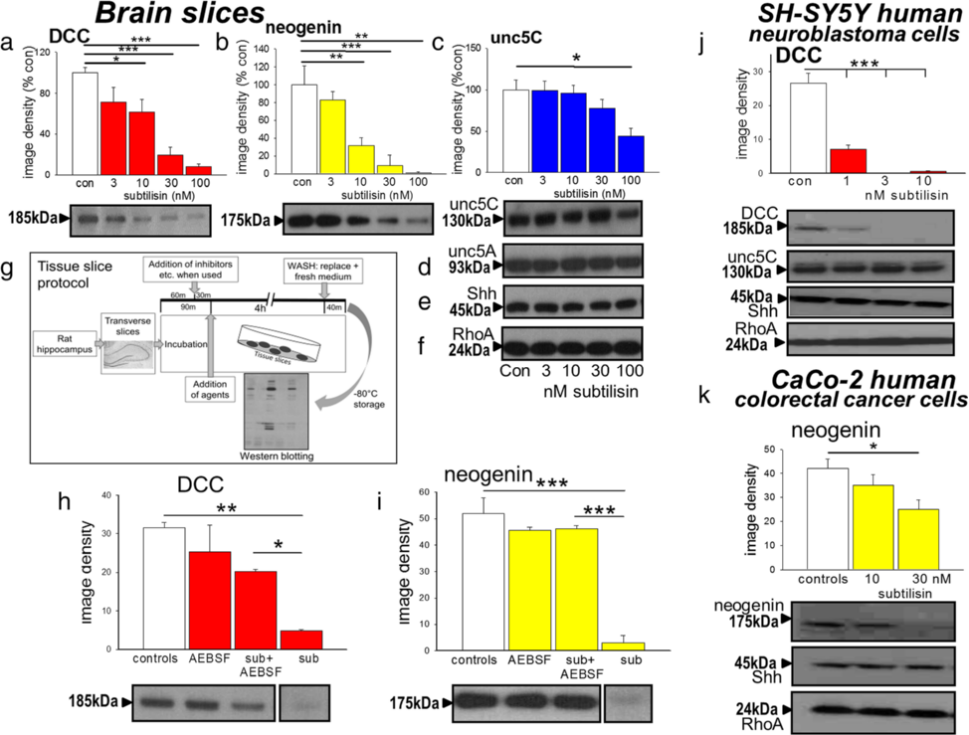
Effects of subtilisin on protein expression. Protein expression in extracts of brain slices is summarised as image densities (arbitrary units) of Western blots quantified using Image J for the effects of subtilisin on (**a**) DCC (**b**) neogenin, (**c**) Unc-5C, (**d**) Unc-5A, (**e**) Shh and (**f**) RhoA expression. Sample blots are shown below each chart (**a**-**c**), which illustrate the concentration-dependent effects of subtilisin and the selectivity of its effects. Scheme (**g**) is a graphic summary of the experimental protocol for these and subsequent experiments. Panels (**h**) and (**i**) summarise the blockade by AEBSF (100 μM, 4 h) of the depletion of DCC and neogenin by subtilisin (sub, 30nM), confirming the role of serine protease activity. Panel (**j**) summarises the effects of subtilisin (1, 3 and 10nM) on the expression of DCC (chart and blot) and on unc-5C, Shh and RhoA (blots) after 7 days in cultures of SH-SY5Y cells. Human colorectal cancer CaCo-2 cells showed a similar susceptibility with reduced expression of neogenin and unc-5C (**k**) but no change in RhoA or Shh after 7days in subtilisin at 10 or 30nM. Bars represent mean ± s.e.mean (*n* = 4). *P < 0.05; **P < 0.01; ***P < 0.001 relative to the control bar

An inverse, concentration-related effect of bacterial subtilisin was observed on the expression of both DCC and neogenin in tissue slices (Fig. 1a,b). A weaker effect was noted on the other netrin receptor unc-5C (Fig. 1c) but no change was seen in the expression of Unc-5A (Fig. 1d), sonic hedgehog (Shh, Fig. 1e) or RhoA (Fig. 1f). To confirm that these effects of subtilisin were mediated by serine protease activity it was shown, using the protocol in Fig. 1g, that they were prevented by treatment with the general serine protease inhibitor 4-(2-Aminoethyl) benzene-sulfonyl fluoride hydrochloride (AEBSF) (Fig. 1h, i).

The loss of dependence receptor proteins was confirmed using cell cultures which lack the tissue barriers and non-selective binding sites that exist in intact tissue. When added to SH-SY5Y cultures for 7 days, subtilisin induced a significant loss of DCC expression at concentrations of only 1nM (Fig. 1j). The selectivity of action was retained and no changes were seen in unc-5C, Shh or RhoA (Fig. 1j).

The ability of subtilisin to deplete neogenin or DCC is not confined to neural tissues. Human CaCo-2 colorectal cancer cells do not express DCC but levels of both neogenin and unc-5C were reduced by subtilisin at 30nM (Fig. 1k) with no effect on RhoA or Shh (Fig. 1k).

**Fig. 2:**
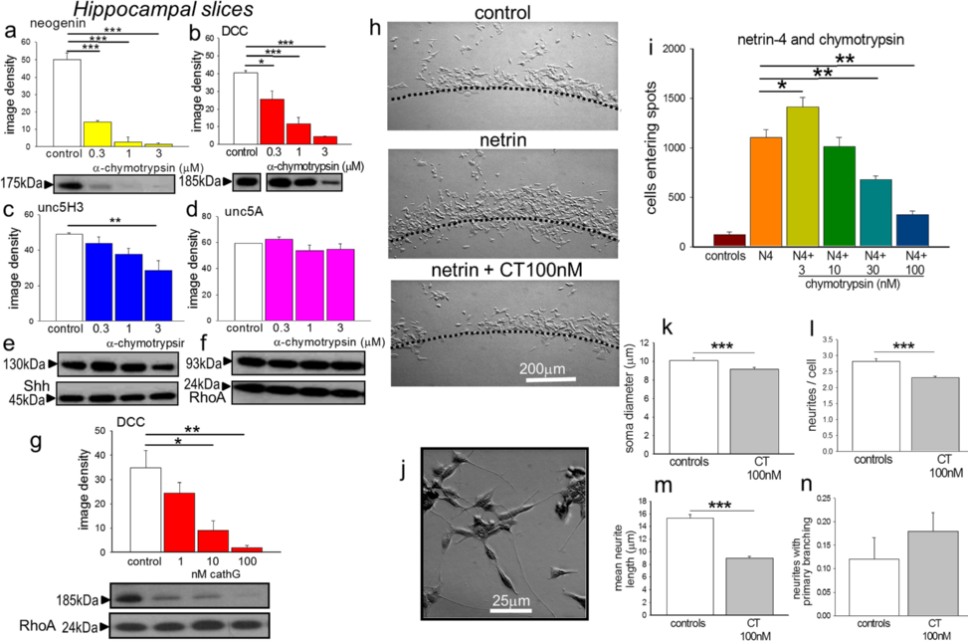
Effects of chymotrypsin on protein expression. Protein expression in extracts of brain slices is summarised as image densities (arbitrary units) of Western blots quantified using Image J for the effects of chymotrypsin on (**a**) neogenin, (**b**) DCC, (**c**) Unc-5C, (**d**) Unc-5A, (**e**) Shh and (**f**) RhoA expression. Sample blots are shown below each chart, which illustrate the concentration-dependent effects of chymotrypsin. Chart (**g**) shows the depletion of DCC by the chymotryptic protein cathepsin G in tissue slices. Panels (**h**) illustrate the agarose spot assay to study the migration of SH-SY5Y cells towards netrin-4. The spot border is indicated by the dotted line. The accumulation of cells on the outside of a normal spot is shown in the top micrograph (control), with the penetration of cells into the spot induced by netrin-4 (netrin, middle micrograph) and reduced penetration in the presence of chymotrypsin (netrin + CT 100nM). The number of cells entering the spots is summarised in chart (**i**). Photograph (**j**) is of SH-SY5Y cells used for the measurement of neurite growth after staining with cresyl violet, with bipolar and multipolar cells visible. The accompanying bar charts summarise the measurements of soma diameter (**k**), neurites per cell (**l**), mean neurite length (**m**) and the proportion of neurites with primary branching (**n**). Bars in charts (**a**) to (**i**) represent mean ± s.e.mean (*n* = 4). *P < 0.05; **P < 0.01; ***P < 0.001 relative to the control bar. Calibration bars: 200 μm in (**h**), 25 μm in (**j**)

Consistent with the classification of subtilisin as a chymotryptic serine protease, chymotrypsin itself also depleted neogenin and DCC in brain tissue (Fig. 2a, b) with a weaker effect on unc-5C (Fig. 2c) and no effect on Unc-5A (Fig. 2d), Shh (Fig. 2e) or RhoA (Fig. 2f). On SH-SY5Y neuroblastoma cells, chymotrypsin produced a similarly selective loss of DCC expression at concentrations similar to those active in intact adult tissue, with approximately 50 % loss of DCC at 300nM. The chymotrypsin-like enzyme cathepsin G, secreted by neutrophils and mast cells as part of the inflammatory response, showed a similar effect, reducing DCC protein expression at concentrations of 10nM or above (Fig. 2g).

### Cell migration

In addition to its role in cell survival, netrin provides a chemoattractive stimulus for the movement of cells. The attraction is mediated by netrin binding to DCC or neogenin and is increased by over-expression of these receptors and prevented in some tissues by DCC antibodies or anti-sense RNA [73, 74].

Cell migration studies using netrin incorporated into an agarose gel spot, showed that SH-SY5Y neuroblastoma cells moved towards netrin (Fig. 2h,i) over a period of 24 h [59]. Since the levels of DCC are low in cancer-derived cell lines, netrin-4 was used in these experiments, since this is an effective ligand for neogenin, which is abundant in SH-SY5Y neuroblastoma cells. In the presence of netrin-4, the number of cells reaching the agarose spot increased approximately 10-fold relative to control wells (Fig. 2i). The addition of chymotrypsin at 3nM produced a small enhancement of migration, while at 10nM or above chemotaxic migration was inhibited (Fig. 2i) consistent with previous data [36] and with its suppression of DCC and neogenin expression. The initially increased attraction probably results from the relative amounts of DCC and neogenin in the cells since neogenin is a target of both netrin and the Repulsive Guidance Molecules. Since chymotrypsin is slightly more potent in reducing neogenin than DCC expression (Fig. 2a,b), this would unmask the net attractive effect of DCC in the absence of neogenin.

These effects of chymotrypsin were associated with changes in neuronal morphology. DCC and neogenin are involved in neurite or growth cone formation and collapse essential for axonal guidance [74–76]. When exposed to chymotrypsin at 100nM for up to 72 h, SH-SY5Y cells (Fig. 2j) exhibited a significant reduction in soma diameter (Fig. 2k), the number of neurites per cell (Fig. 2l) and the mean length of neurites (Fig. 2m). There was also a trend to increase neuritic branching (Fig. 2n). Such changes in neuritic parameters are consistent with the proposed involvement of DCC and neogenin in the regulation of neurite elongation and branching [73, 77, 78] and support the concept that the depletion of dependence receptors by subtilisin and chymotrypsin has the expected functional consequences.

### Wound healing assays

**Fig. 3:**
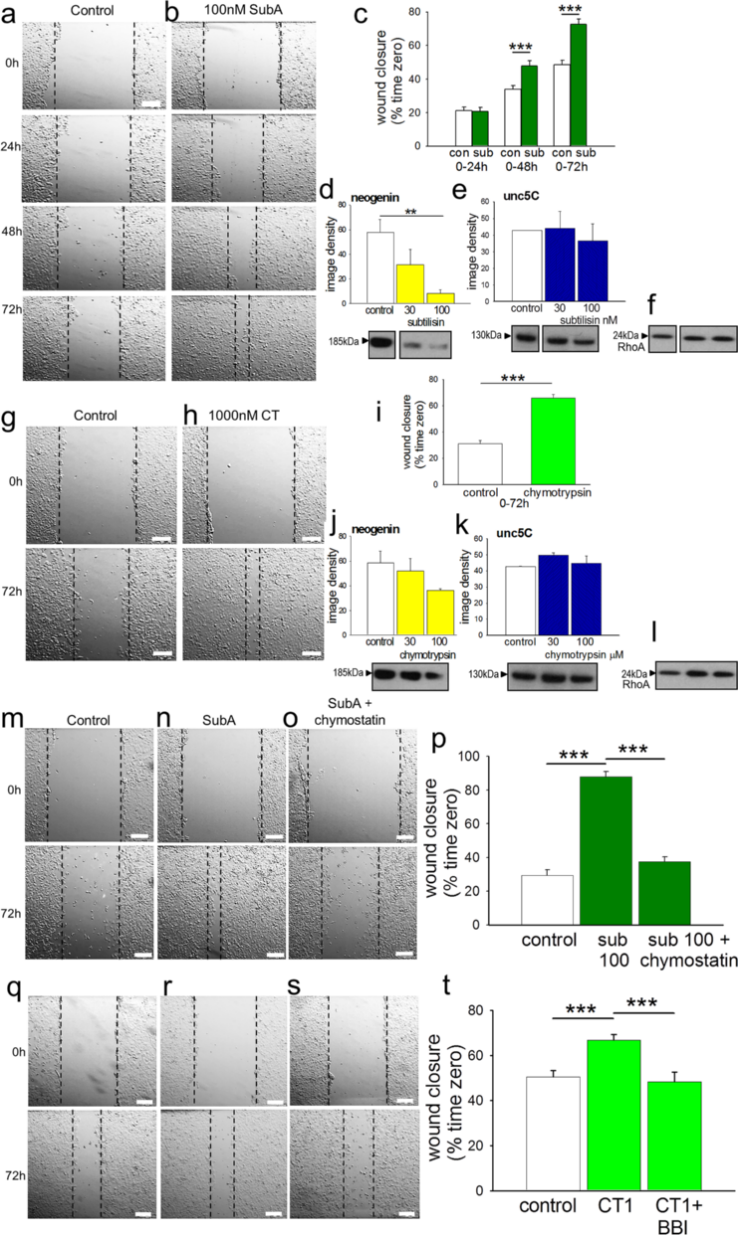
Effects of serine protease inhibitors on wound closure. At 100nM subtilisin promotes the closure of a scratch wound in MCF-7 cultures between 0 h and 72 h relative to the closure rate in untreated cells (**a**, **b**). The quantified scratch closure data are summarised in chart (**c**). The effect of subtilisin at 30 and 100nM is shown on the expression of (**d**) neogenin, (**e**) unc-5C, (**f**) RhoA in the same cell line of MCF-7 breast adenocarcinoma cells. Chymotrypsin (1 μM) shows a similar ability to facilitate wound closure (**g**, **h**) as summarised in panel (**i**). Chymotrypsin has a similar but weaker action on neogenin expression (**j**) without affecting unc-5C (**k**) or RhoA (**l**). In separate experiments (**m**) the rate of wound closure is again facilitated by subtilisin (**n**) whose effect is blocked by the chymotryptic protease inhibitor chymostatin (**o**, **p**). Similarly the Bowman-Birk inhibitor from soybean was able to completely block the facilitation of cell migration produced by chymotrypsin (**q**-**t**). Bars represent mean ± s.e.mean of the percentage change in wound area (*n* = 3–4). **P < 0.01; ***P < 0.001 relative to the control bar. Calibration bars: 200 μm

Migration was also studied using MCF-7 cells in the wound or scratch assay [60, 61], MCF-7 cells exhibit a lower basal level of motility than MDA-MB-231 cells and the latter line includes significant numbers of floating cells which complicate interpretation of the results. At 100nM, subtilisin increased the rate of closure of the scratch wound compared with control cells over 72 h (Fig. 3a-c). Protein expression in these cells showed sensitivity to subtilisin and chymotrypsin similar to that of the adult brain tissue and neuroblastoma cells, with subtilisin at 100nM producing a substantial reduction in neogenin expression (Fig. 3d) but no change of unc-5C or RhoA expression (Fig. 3e, f).

Chymotrypsin showed a similar ability to facilitate wound closure over 72 h (Fig. 3g-i). As in the preceding work, chymotrypsin (1 μM) was less effective than subtilisin but still reduced neogenin expression by 40 % compared with control cultures after 48 h exposure (Fig. 3j) while having no effect on unc-5C (Fig. 3k) or RhoA (Fig. 3l).

The involvement of chymotryptic protease activity in the effect of subtilisin was confirmed by showing that chymostatin, the most selective chymotryptic inhibitor available, could prevent the facilitation of wound closure by subtilisin (Fig. 3m-p).

Similarly, the Bowman-Birk inhibitor from soybean (see below) at the relatively low concentration of 50 μM was able to block completely the facilitation of wound closure by chymotrypsin (Fig. 3q-t). This concentration was used since higher levels can directly affect cell migration, complicating interpretation of the results. Even at this low concentration, however, the inhibitor showed a strong tendency to block the promotion of wound closure produced by subtilisin, which just failed to reach statistical significance (*P* = 0.06) (data not shown).

**Fig. 4:**
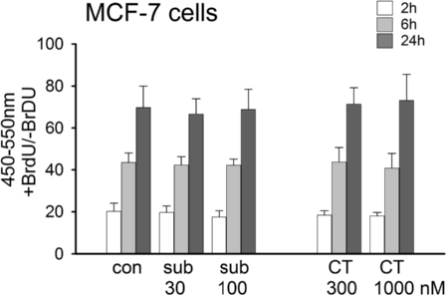
Proliferation of MCF-7 cells. Using the BrdU for cell proliferation, neither subtilisin 30 or 100nM, nor chymotrypsin 300 or 1000nM had any effect on the proliferation of MCF-7 breast cancer cells after 2, 6 or 24 h incubation with the protease (*n* = 3)

Cell proliferation was examined using the bromodeoxyuridine (BrdU) uptake method. After 2 h, 6 h or 24 h exposure neither subtilisin (30 or 100nM) nor chymotrypsin (300 or 1000nM) had any effect on the proliferation of MCF-7 cells (Fig. 4), indicating that changes in proliferation could not account for their efficacy on wound healing.

### Reversibility of cell behaviour

**Fig. 5:**
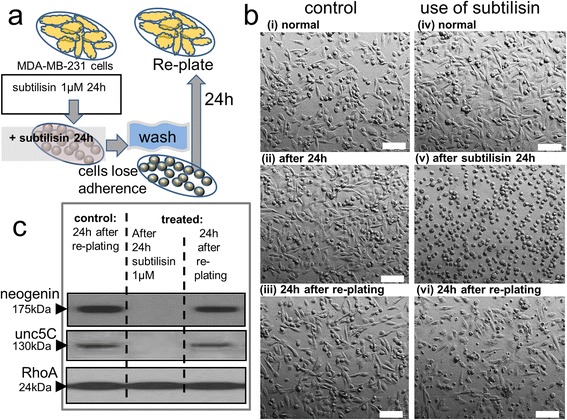
Reversibility of subtilisin. Panel (**a**) illustrates the experimental paradigm. MDA-MB-231 cells, as a mixture of flattened and rounded cell types attached to the dish together with some detached, floating cells (**b**(i),(iv)), were treated with a relatively high concentration of subtilisin (1 μM) for 24 h. This caused most cells to detach from the culture plate and appear as spherical cells floating in the culture medium either as individual cells (**b**(v)) or as aggregates. After 24 h, the cells were washed in fresh medium and transferred to new wells, where the detached cells re-attached to the well surface and grew to reform the apparently normal mixture of flat and rounded, adherent cultures (**b**(vi)). Control cells were treated similarly by washing (**b**(ii)) and transference to new wells (**b**(iii)) in parallel with the subtilisin-treated dishes. The ability to recover from subtilisin treatment was supported by protein expression (**c**). There was a substantial reduction in the expression of neogenin and unc-5C, with preservation of RhoA levels (**c**) in the presence of subtilisin. When the cells were washed and re-plated as above, expression of these proteins recovered to their orginal levels within 24 h, in parallel with the recovery of normal cell morphology. Scale bar: 100 μm

An important property of many cells in culture is anoikis – the induction of apoptosis when they become detached from a tissue or artificial substratum [79]. This behaviour mimics the in vivo propensity of cells to undergo apoptosis if they become detached from their home tissue, a phenomenon thought to protect organisms by preventing the migration of potentially abnormal cells to a distant site where they may become overtly oncogenic and establish metastases. To examine the effects of serine proteases we used the relatively aggressive human breast adenocarcinoma cell line of MDA-MB-231 cells. These cells normally exist as a mixture of flattened, multipolar or spindle-shaped adherent cells (Fig. 5a, b(i)-(iv)) with some detached and floating cells. When subtilisin was added to cultures for 24 h at concentrations of 1 μM, a large proportion of cells detached from the culture plate and appeared as spherical cells floating in the medium, mostly as individual cells (Fig. 5b(v)), which could be maintained for up to 24 h (in 15 % serum). When washed in fresh medium and transferred to new wells within 24 h, the cells re-attached once more and then grew and divided as apparently normal cells, reforming the normal mixture of flat and rounded, adherent cultures (Fig. 5b(vi)). These observations indicate that the exposure of cells to subtilisin for 24 h did not produce irreversible cell toxicity, permanent damage or death and that the loss of adhesion was an acute and reversible phenomenon.

These observations were further supported by an examination of protein expression. In the presence of subtilisin there was a loss or profound reduction in the expression of neogenin and unc-5C, with preservation of RhoA levels (Fig. 5c), but after washing and re-plating, protein expression recovered to their original levels within 24 h, in parallel with the recovery of normal cell morphology (Fig. 5c). These results agree with previous data showing that mutational silencing or deletion of DCC or neogenin using siRNA techniques can produce a loss of cell adhesion leading to detachment [80, 81] supporting the concept that biologically relevant serine proteases can have the same net effects as this highly selective experimental inactivation protocol. Most importantly, the reversibility of cell detachment and dependence receptor depletion by serine proteases reinforces the potential relevance of our observations to cancer cell dispersion and formation of metastases.

### Down-regulation of ectopic DCC

**Fig. 6:**
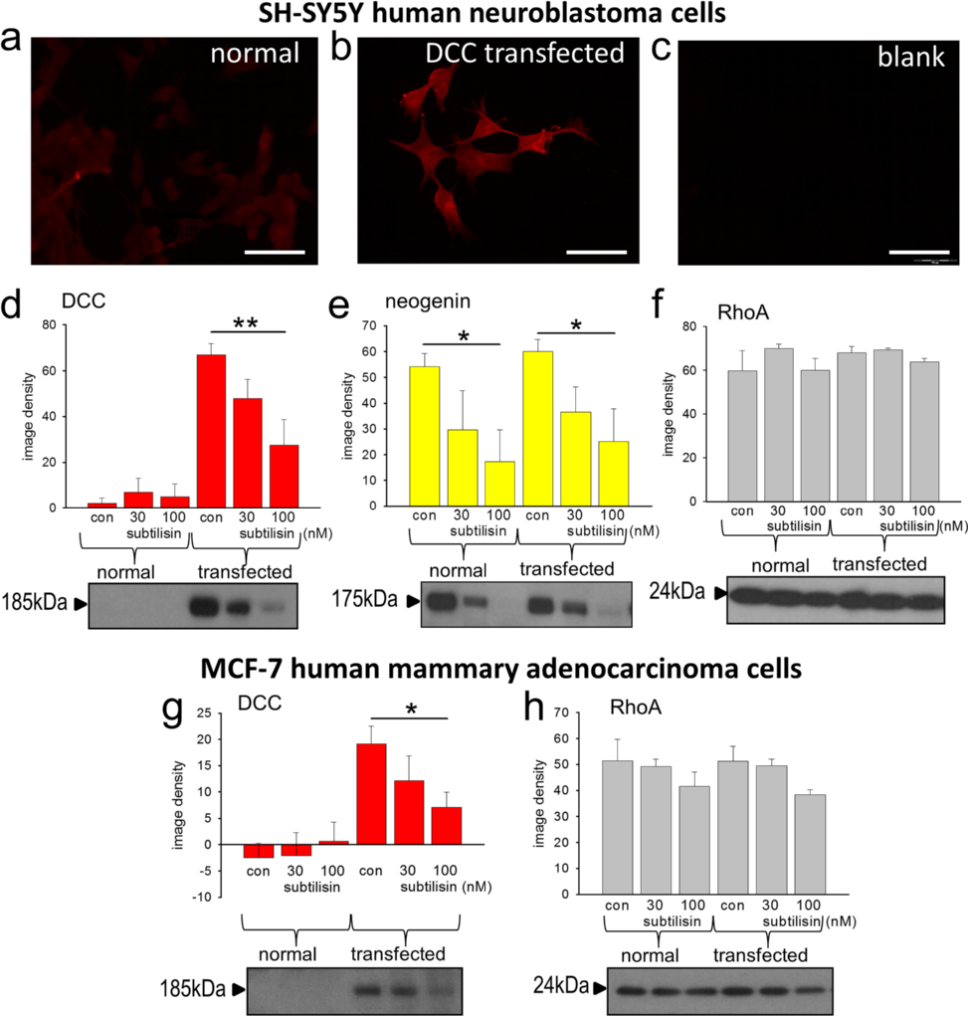
Transfection of dcc into cultured cells. A photomicrograph of normal SH-SY5Y cells after incubation with antibodies to DCC showed only very weak immunofluorescence (**a**) whereas 48 h after transient transfection with a *dcc* plasmid, the fluorescence was substantially greater (**b**) and no image was obtained in the absence of DCC antibody (**c**). The fluorescence images are consistent with immunoblots for DCC protein which indicated an approximately 50-fold increase in expression (**d**). The protein was down-regulated by subtilisin (**d**) (30 and 100nM) as observed previously in adult tissue. The relative expression of neogenin (**e**) or RhoA (**f**) was unchanged by *dcc* transfection and neogenin was reduced by subtilisin while RhoA remained unaffected, as in the tissue work. An exactly similar pattern was seen in transiently transfected MCF-7 cells, with a profound reduction by subtilisin of ectopic DCC expression (**g**) and no change in RhoA (**h**). Bars represent mean ± s.e.mean (*n* = 4–6). *P < 0.05; **P < 0.01; relative to the corresponding control bar. Scale bar: 50 μm

While tissue slices expressed high levels of DCC and neogenin, the cancer-derived cell lines showed little or no intrinsic DCC expression. We therefore inserted the *dcc* gene into SH-SY5Y and MCF-7 cells to generate a population of transiently transfected cells which exhibited much stronger fluorescence than control cells (Fig. 6a-c). Western blots confirmed the very low levels of DCC in naïve cells with approximately 25-fold higher expression in the transfected cells (Fig. 6d). Levels of neogenin and RhoA were not different between control and transfected cells (Fig. 6e, f) confirming that they were not directly affected by the introduction of DCC. The addition of subtilisin (30 or 100nM for 48 h) showed a clear suppression of both ectopic DCC expression (Fig. 6d) and of endogenous neogenin (Fig. 6e) with no effect on RhoA (Fig. 6f). Comparable results were obtained using transfected MCF-7 cells, with a reduction in ectopic DCC expression at 100nM subtilisin (Fig. 6g), but no change in RhoA (Fig. 6h).

### Bowman-Birk and other inhibitors

**Fig. 7:**
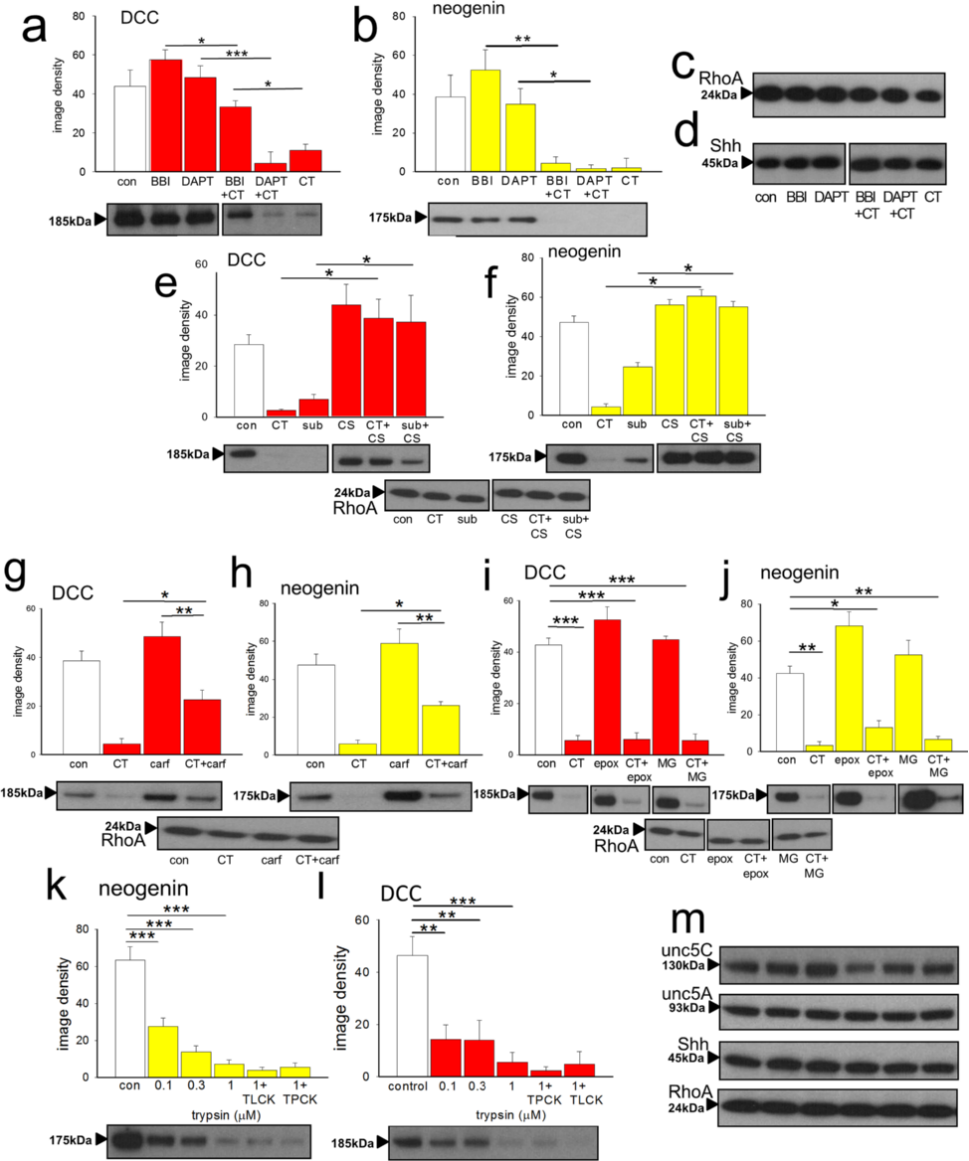
Potential inhibitors of serine proteases. Protein expression in extracts of brain slices is summarised as image densities (arbitrary units) of Western blots quantified using Image J for the effects of chymotrypsin (CT, 1 μM) on DCC (**a**), neogenin (**b**), RhoA (**c**) and Shh (**d**) expression. Sample blots are shown below each chart, which illustrate the concentration-dependent effects of the proteases. The Bowman-Birk soybean inhibitor (BBI, 100 μM) blocked the effect of chymotrypsin on DCC expression (**a**) although the γ-secretase inhibitor DAPT (5 μM) did not prevent the loss of DCC (**c**) or neogenin (**d**). Neither BBI nor DAPT had any effect themselves on protein expression. Chymostatin (CS, 30 μM) had no significant effect alone but blocked the inhibitory effects of both chymotrypsin and subtilisin on DCC (**e**) and neogenin (**f**) expression. The chymotryptic proteasome inhibitor carfilzomib (carf, 50nM) significantly reduced the effect of chymotrypsin (CT, 1 μM) on DCC (**g**) and neogenin (**h**) expression. However, two other inhibitors of the 20S proteasome, epoxomicin (epox, 1 μM) and MG132 (MG, 10 μM) had no significant effect themselves and did not block the effect of chymotrypsin on DCC (**i**) or neogenin (**j**). Trypsin reduced the expression of neogenin (**k**) and DCC (**l**) with no significant effects on unc5H3, unc5H1, Shh and RhoA expression (**m**) but these effects were not prevented by TPCK or TLCK. Sample blots are shown below each chart. Bars represent mean ± s.e.mean (*n* = 4). *P < 0.05; **P < 0.01; ***P < 0.001 relative to the control bar

Families of Bowman-Birk serine protease inhibitors have been isolated from a wide range of plants, including the soybean inhibitor from *Glycine max* [48–51]. They have anti-cancer properties associated with their inhibitory activity against trypsin-like and chymotrypsin-like enzymes [51, 82]. We found that the soybean protease reduced significantly the loss of DCC produced by chymotrypsin (Fig. 7a) without affecting the expression of other proteins such as neogenin, RhoA or Shh (Fig. 7b-d).

To examine the effect of the soybean Bowman-Birk inhibitor on a functional system, it was tested on the wound invasion assay. As noted above and shown in Fig. 3q-t, the inhibitor (50 μM) blocked completely the facilitation of wound closure produced by chymotrypsin (1 μM) and substantially reduced the effect of subtilisin (*P* = 0.06).

Although DCC and neogenin can be metabolised by γ-secretase activity [83–85], the γ-secretase inhibitor *N-*[*N*-(3,5-difluorophenacetyl-L-alanyl)]-(*S*)-phenylglycine-*t*-butyl ester (DAPT; 5 μM) did not modify the basal expression of DCC or neogenin or their depletion by chymotrypsin (Fig. 7a-d). The bacterial chymotryptic inhibitor chymostatin blocked the effects of chymotrypsin and subtilisin on DCC (Fig. 7e) and neogenin (Fig. 7f). Selective inhibitors of proteasomal chymotryptic activity – carfilzomib [86–88], epoxomicin (epox, 1 μM) [89, 90] and carbobenzoxy-Leu-Leu-leucinal (MG132, 10 μM) [91] - did not affect DCC or neogenin expression (Fig. 7g-j) although carfilzomib did reduce significantly the effect of chymotrypsin.

Another endogenous serine protease, trypsin, also reduced the expression of neogenin (Fig. 7k) and DCC, (Fig. 7l) with no significant effects on Unc-5C, Unc-5A, Shh and RhoA expression (Fig. 7m). These effects were not prevented by N-α-tosyl-L-lysine-chloromethyl-ketone (TLCK) - an inhibitor of trypsin-like enzymes, or the general inhibitor of chymotryptic proteases, N-tosyl- L-phenylalanyl-chloromethyl-ketone (TPCK) (Figs. 7k, l).

TPCK, but not TLCK, blocked the effect of chymotrypsin on neogenin expression (Additional file 1: Figure S1a, b) and increased DCC to a level not significantly different from controls (Additional file 1: Figure S1b). Neither TPCK nor TLCK blocked the effects of subtilisin on DCC or neogenin (Additional file 1: Figure S1c, d) indicating that subtilisin is atypical, despite its blockade by chymostatin (see Fig. 7e, f).

Although DCC and neogenin can also be degraded by matrix metalloprotease-9 (MMP-9) [83, 84, 92], a selective inhibitor of MMP-9 (2-((4-phenoxyphenylsulfonyl)-methyl) thiirane); SB-3CT) did not alter expression of the dependence receptors or prevent their down-regulation by chymotrypsin (Additional file 1: Figure S2A, B). The non-selective MMP inhibitor marimastat (10 μM) was also ineffective in blocking the protease activity (Additional file 1: Figure S2a,b).

## Discussion

The present results reveal the ability of endogenous chymotrypsin and an environmental, bacterial chymotryptic protease, subtilisin, to down-regulate DCC and neogenin expression, increasing cell migration. The reversibility of the changes in adhesion and protein expression would be relevant for cells to migrate and form metastases elsewhere. Other proteins with strong functional links with DCC were unaffected.

Even in prokaryotes, serine proteases are involved in the control of proliferation and modulation of the cell cycle [93] and an association with cancer was recognised some years ago [94–98]. Chymotrypsin and related enzymes increase proliferation and migration [35, 99–104] and high levels occur in several cancers, especially in mammary myoepithelial cells and some solid tumours [105–108], correlating with the development of malignant disease. Tumour cell aggression is correlated with serine protease activity in models of carcinogenesis and metastasis [109–111]. Conversely, serine protease inhibitors or enzyme down-regulation reduce cell migration and invasiveness [82, 112–114] while reduced levels of serine protease inhibitors promote oncogenesis [42]. Trypsin is also linked with the development of cancers [36] including pancreatic adenocarcinoma [115] and colorectal cancer, cases of the latter showing poor patient prognosis correlating with their trypsin content [116, 117]. Indeed, serine proteases are involved in a wide range of biological activities [118] including embryonic development, osteogenesis, and immune cell function, making the present results potentially of far wider physio-pathological significance than only for cancer.

### Dependence receptors

The experimental deletion or disruption of the *dcc* gene can induce or promote cell migration and invasiveness [11–14] while transfecting cells with the gene can reduce these characteristics [13–17, 80]. Neogenin expression is also reduced in tumours, especially those involving mammary tissue [20, 25], where it regulates cell proliferation, migration and invasion [119]. Over-expression can suppress abnormal proliferation and migration [22, 23]. The third netrin receptor (unc-5C) is also involved in cell proliferation and migration and is reduced in many cancers [33, 46, 64, 120]. However, since there are few mutations of the respective genes in cancerous cells, non-genetic modifications may be sufficient to cause cellular dysfunction [9, 10], a conclusion consistent with growing evidence that non-genetic factors play a dominant role in oncogenesis [121–123]. The down-regulation of DCC and neogenin by serine proteases could be one example of the several processes involved [58].

### Chymotrypsin and cancer

**Fig. 8:**
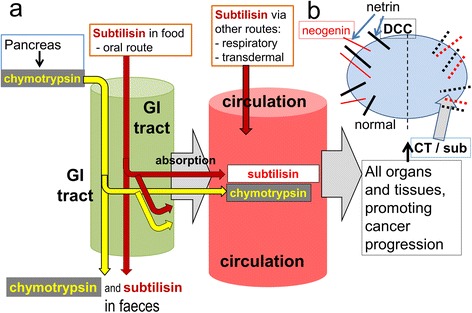
A schematic summary of the hypothesis. **a** The pancreas secretes chymotrypsin (yellow) as a digestive enzyme in proportion to the amount of food (protein) consumed. Most passes through the gastro-intestinal (GI) tract unchanged. Subtilisin present in processed foods as well as *Bacillus subtilis* present in soil, livestock feed and probiotics, enter the gastro-intestinal tract orally, while subtilisin present in cleaning products may access the body via the respiratory system or dermal contact. Both proteases will be in contact with cells in the intestinal epithelium and some will be absorbed into the cardiovascular circulation from where both will access all organs and tissues, promoting oncogenesis. **b** At the cellular level netrin promotes cell stability and proliferation, with DCC acting to inhibit that activity. The unhindered activity of DCC induces cell death, partly by apoptosis. When DCC is removed by subtilisin or chymotrypsin, netrin can promote proliferation leading potentially to cancer

Chymotryptic activity is present in the blood and other tissues in the form of chymotrypsin and related proteins such as chymase and cathepsins released from neutrophils during inflammation. Chymotrypsin can also be absorbed, along with other large proteins, from the intestinal contents into the blood [124]. As a result these endogenous proteases are in direct contact with most organs and tissues (Fig. 8). Chymotrypsin is resistant to destruction in the gut in order to carry out its digestive functions and its concentration in the intestinal contents changes little from pancreas to faeces, enabling faecal chymotrypsin concentrations to be used as an indicator of pancreatic function [125–128]. The concentrations of chymotrypsin in normal human chyme and faeces are around 1-10 μM [129–131], levels similar to, and often higher than those able to deplete neogenin and DCC. It is likely, therefore, that this depletion also occurs in vivo, its impact normally limited by the continual replacement of intestinal epithelial cells or a plant-rich diet. Slowed transit times or the regular consumption of protease-treated meat products may increase the impact of chymotryptic activity on dependence receptor expression.

Chymotrypsin production is increased in obese individuals, partly in proportion to food volume in order to process the protein content and partly in response to a raised consumption of meat, which has a higher proportion of protein per unit weight than dietary plants. Changing to a vegan diet reduces the secretion of chymotrypsin and the intestinal concentration [126], consistent with the lower colorectal cancer levels in vegetarians [132].

### Subtilisin, diet and cancer

Subtilisin is a chymotryptic serine protease synthesised and secreted by *B. subtilis* and related species. Closely related enzymes with similar substrate specificities are secreted by other bacteria (*Streptomyces spp* and *Cryptosporidium spp*) [133–135] as well as some fungi and yeasts (*Aspergillus spp* [136, 137], *Cryphonectria parasitica* [138] and *Trichoderma reesei*), the latter secreting particularly large quantities of a subtilisin-like protease [139].

*B. subtilis* itself is ubiquitous in the environment and in high densities in soil. It is a normal commensal bacterium in the mammalian intestine [47, 140, 141] since the bacteria and spores resist destruction in the stomach and intestine [142] (Fig. 7). The bacteria are included in many probiotic preparations for use in humans or farming livestock to promote digestion and to increase muscle mass [140, 143–147] (approximately 1,000 tonnes of the enzyme are used annually in Europe alone [148]). High densities of the bacteria are observed in abattoirs and food-processing plants [149, 150] where it is sometimes used to tenderise meats and to promote compaction after butchering. Since the bacteria are highly resistant to acidity or temperature changes (including boiling), subtilisin of bacterial or environmental origin which enters the food chain represents a candidate for mediating the effects of diet and environment on cancer generation by depleting cellular DCC and neogenin. Being of similar molecular size to chymotrypsin and smaller then ferritin, subtilisin is also likely to be absorbed from the intestine [124] into the systemic blood from where it would reach all the organs and tissues and potentially promote cancer development at those sites.

The enzyme is also used in some cleaning preparations and exfoliants. The US Household Products Database catalogues more than 100 domestic cleaning products which contain subtilisin [151] and others are available elsewhere. Such sources represent a further route of biological access for subtilisin in the industrial and domestic environments.

### Protease inhibitors

The case for a serine protease involvement in cancer (Fig. 8) is strongly supported by the prevention of dependence receptor loss by a Bowman-Birk inhibitor. These compounds are selective inhibitors of serine proteases [152] and are produced by many species of plant including soybeans, lentils [48], wheat [153], potatoes [49, 154] and other sources [50, 155]. They are highly resistant to heat and metabolism, passing largely unchanged through the gut after dietary consumption. Importantly, the inhibitor was also able to prevent the increase in cell migration produced by chymotrypsin in the wound assay, and partly reduced the effect of subtilisin when used at a low concentration. These actions would be consistent with the protective anti-cancer effects of a plant-rich diet. This may be especially important in preventing metastasis formation since this is dependent on serine proteases released from neutrophils which have been attracted to areas of inflammation [156] and which are inhibited by Bowman-Birk inhibitors [157]. These inhibitors have been shown previously to have anti-cancer activity in vitro [158, 159] and in humans and other animals. After oral administration they can suppress the development not only of intestinal cancers [160, 161] but also those in other tissues after absorption from the gut [162–165]. A concentrated plant extract of Bowman-Birk inhibitors has proved effective in human clinical trials [51], although a precise site of action had not previously been identified [166].

### Cancer prevention and public health

Further work is required to establish the precise relationship between continually elevated concentrations of subtilisin and chymotrypsin in the blood after chronic, continual, oral administration, their selective removal of dependence receptors, and the development of cancers in vivo, especially in relation to the development of late-stage and malignant disease. In addition to providing an explanation of a major environmental influence on cancer development, these relationships might justify the clinical use of protease inhibitors in combination with existing radiotherapy and chemotherapy. Thus, if the presence of subtilisin in the environment and food chain could be reduced so that the progression of tumours to malignancy was slowed or prevented, conventional agents should be able more effectively to produce a complete elimination of the disease.

Overall, our results suggest the hypothesis that lifestyle factors such as food choice may make a contribution to cancer incidence and malignancy [122]. They also suggest a socio-economically valuable public health strategy. Eliminating the use of subtilisin in farming livestock and meat processing, domestic cleaning preparations and other sources in the environment, encouraging careful washing of crops and increasing the dietary intake of plant-sourced serine protease inhibitors, could potentially reduce the worldwide incidence of several forms of cancer by reducing serine protease-induced removal of DCC and neogenin.

## Conclusions

The present results show that two representative serine proteases, endogenous mammalian chymotrypsin and an environmental, bacterial chymotryptic protease, subtilisin, can down-regulate DCC and neogenin expression in cells, increasing cell migration. The reversibility of the changes in adhesion and protein expression would be important for cells which migrate from their home tissue to form metastases elsewhere. Since chymotrypsin secretion is increased by over-eating and increased basal metabolic rate, and it can be absorbed from the intestine where is exists as a normal digestive enzyme, it may provide an explanation of the link between over-eating and cancer incidence. Subtilisin is used in meat tenderisation and processing, and domestic and industrial cleaning products, while its main producer, the bacterium *B. subtilis*, is added to probiotics and food for farm animals to promote growth. This relationship may contribute to the link between meat consumption and cancer incidence, while inhibition of chymotryptic enzymes by Bowman-Birk inhibitors from plants may explain the protective effects of a plant-based diet. The data reported here, therefore, may help understanding of the causes of many cancers, with the potential to prevent many of them by restricting the industrial and agricultural use of serine proteases. In addition, further work on the efficacy of dietary Bowman-Birk compounds as inhibitors of subtilisin and chymotrypsin might lead to their increased use as protection against cancer.

## Additional file

Additional file 1: Supplementary material. Supplementary Figures S1, S2 and associated legends. (DOC 900 kb)

## Abbreviations

AEBSF: 4-(2-Aminoethyl) benzenesulfonylfluoride hydrochloride (AEBSF)
AYPGKF-NH2: Ala-tyr-pro-gly-lys-phe-NH2 trifluoroacetate (Sigma A3227)
BBI: Bowman-Birk protease inhibitor
BrdU: Bromodeoxyuridine
CSF: Cerebrospinal fluid
DAPT: *N-*[*N*-(3,5-Difluorophenacetyl-L-alanyl)]-(*S*)-phenylglycine *t*-butyl ester
DCC: Deleted in colorectal cancer
DR(s): Dependence receptor(s)
MAR: Marimastat
MG132: Carbobenzoxy-Leu-Leu-leucinal
MMP: Matrix metalloprotease
RhoA: Ras homologue A
SB-3CT: 2-((4-phenoxyphenylsulfonyl)methyl) thiirane
Shh: Sonic hedgehog
TLCK: N-alpha-tosyl-L-lysine-chloromethylketone
TPCK: N-tosyl- L-phenylalanyl-chloromethyl-ketone (α-CT specific)
Unc: Unco-ordinated locomotion (unc5-A-D in non-human animals these were formerly referred to as unc5H1-4)
