# Supplementary material for: Selective depletion of tumour suppressors Deleted in Colorectal Cancer (DCC) and neogenin by environmental and endogenous serine proteases: linking diet and cancer
Source: BMC Cancer. 2016 Oct 6;16:772. doi: 10.1186/s12885-016-2795-y (PMC5054602; doi:10.1186/s12885-016-2795-y)

**SUPPLEMENTARY MATERIAL**

**Supplementary Figure Legends**

**Supplementary Figure S1**

***Effects of proteases and inhibitors on dependence receptor expression.***

Protein expression in extracts of brain slices is summarised as image densities (arbitrary units) of Western blots quantified using Image J for the effects of chymotrypsin (CT: A,B) and subtilisin (sub, C,D) on DCC (A,C) and neogenin (B,D) expression. Sample blots are shown below each chart. TLCK (150 µM) increased the resting levels of both DCC and neogenin but did not block the depletion of these proteins by chymotrypsin (A,B) or subtilisin (C,D). TPCK (150 µM) partially blocked the effect of chymotrypsin such that the level of DCC (A) and neogenin (B) expression was not significantly different from control levels but it did not prevent the effects of subtilisin (C,D). Typical sample blots are illustrated below the corresponding bar charts. Bars represent mean ± s.e.mean (n = 3). **P* < 0.05; ***P* < 0.01; ****P* < 0.001 relative to the control bar.

**Supplementary Figure S2**

***Inhibitors of metalloproteinases***

Protein expression in brain slices is summarised as image densities (arbitrary units) of Western blots quantified using Image J for the effects of chymotrypsin (CT, 1µM) on DCC (A) and neogenin (B) expression. Sample blots are shown below each chart. The loss of proteins induced by CT is not prevented by SB-3CT (SB, 5µM, a selective inhibitor of MMP-9), or marimastat (MAR, 10µM), a non-selective inhibitor of most MMP enzymes. The inhibitors were added 30 min before chymotrypsin to ensure complete inhibition of MMP-9 (see Fig. 1G), followed by the addition of chymotrypsin for 4h. Bars represent mean ± s.e.mean (n = 3). ***P* < 0.01; ****P* < 0.001 for CT relative to the control bar or for CT plus inhibitor relative to the inhibitor alone.

Supplementary Figure S1


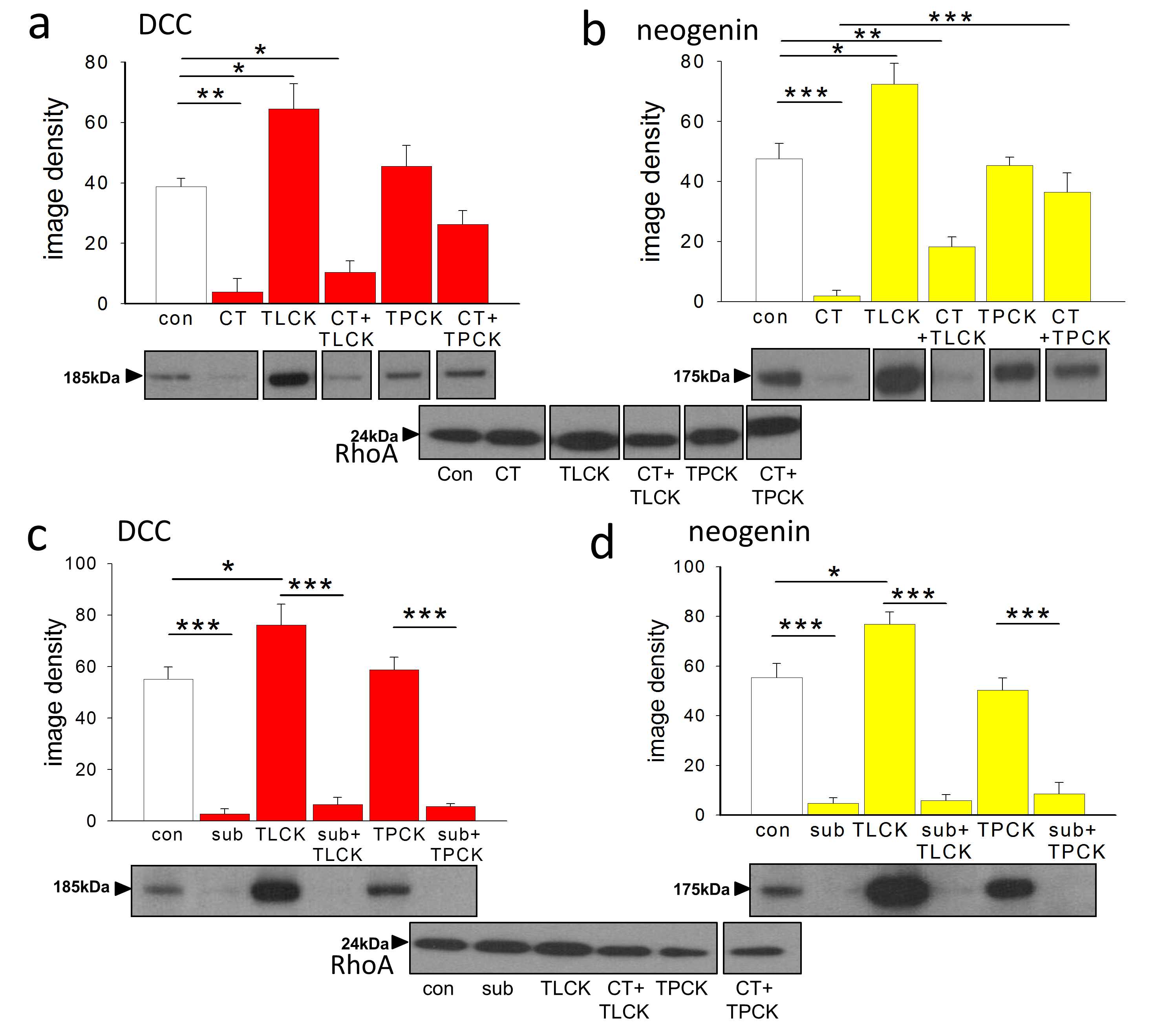


Supplementary Figure S2


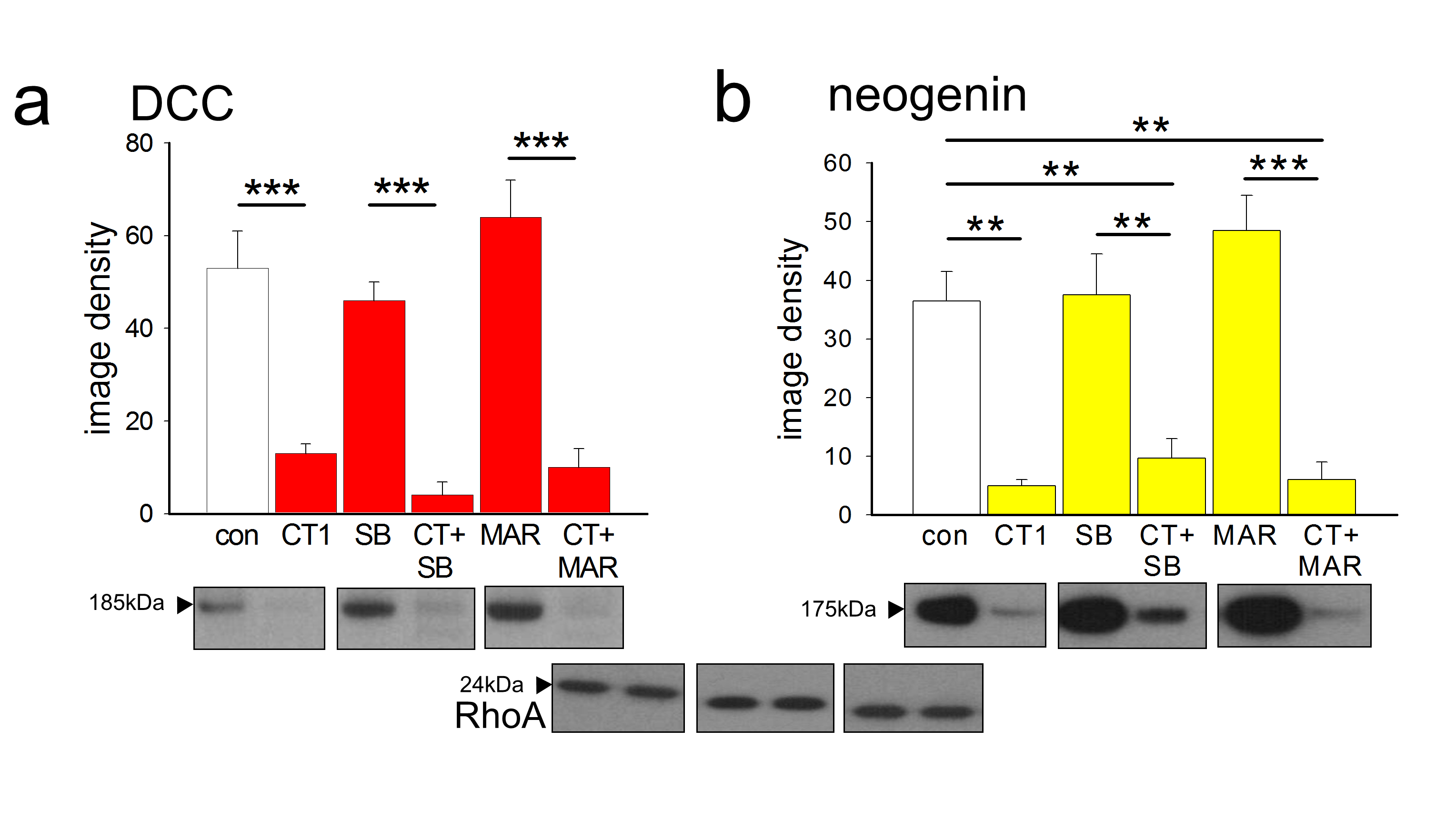

Supplement: Supplementary file 1 — Supplementary material. Supplementary Figures S1, S2 and associated legends. (DOC 900 kb) [file 12885_2016_2795_MOESM1_ESM.doc]
